# Harnessing Clinical Psychiatric Data with an Electronic Assessment Tool (OPCRIT+): The Utility of Symptom Dimensions

**DOI:** 10.1371/journal.pone.0058790

**Published:** 2013-03-08

**Authors:** Philip James Brittain, Sarah Elizabeth Margaret Lobo, James Rucker, Myanthi Amarasinghe, Anantha Padmanabha Pillai Anilkumar, Martin Baggaley, Pallavi Banerjee, Jenny Bearn, Matthew Broadbent, Matthew Butler, Colin Donald Campbell, Anthony James Cleare, Luiz Dratcu, Sophia Frangou, Fiona Gaughran, Matthew Goldin, Annika Henke, Nikola Kern, Abdallah Krayem, Faiza Mufti, Ronan McIvor, Humphrey Needham-Bennett, Stuart Newman, Dele Olajide, David O’Flynn, Ranga Rao, Ijaz Ur Rehman, Gertrude Seneviratne, Daniel Stahl, Sajid Suleman, Janet Treasure, John Tully, David Veale, Robert Stewart, Peter McGuffin, Simon Lovestone, Matthew Hotopf, Gunter Schumann

**Affiliations:** 1 National Institute for Health Research Specialist Biomedical Research Centre for Mental Health at the South London and Maudsley National Health Service Foundation Trust and The Institute of Psychiatry, King's College London, London, United Kingdom; 2 Medical Research Council Social, Genetic and Developmental Psychiatry Centre, Institute of Psychiatry, King’s College London, London, United Kingdom; 3 Institute of Psychiatry, King’s College London, London, United Kingdom; 4 Department of Forensic and Developmental Neuroscience, Institute of Psychiatry, King’s College London, London, United Kingdom; 5 National Psychosis Service, South London and Maudsley National Health Service Foundation Trust, Bethlem Royal Hospital, London, United Kingdom; 6 South London and Maudsley National Health Service Foundation Trust, Bethlem Royal Hospital, London, United Kingdom; University of Melbourne, Australia

## Abstract

Progress in personalised psychiatry is dependent on researchers having access to systematic and accurately acquired symptom data across clinical diagnoses. We have developed a structured psychiatric assessment tool, OPCRIT+, that is being introduced into the electronic medical records system of the South London and Maudsley NHS Foundation Trust which can help to achieve this. In this report we examine the utility of the symptom data being collected with the tool. Cross-sectional mental state data from a mixed-diagnostic cohort of 876 inpatients was subjected to a principal components analysis (PCA). Six components, explaining 46% of the variance in recorded symptoms, were extracted. The components represented dimensions of mania, depression, positive symptoms, anxiety, negative symptoms and disorganization. As indicated by component scores, different clinical diagnoses demonstrated distinct symptom profiles characterized by wide-ranging levels of severity. When comparing the predictive value of symptoms against diagnosis for a variety of clinical outcome measures (e.g. ‘Overactive, aggressive behaviour’), symptoms proved superior in five instances (R^2^ range: 0.06–0.28) whereas diagnosis was best just once (R^2^∶0.25). This report demonstrates that symptom data being routinely gathered in an NHS trust, when documented on the appropriate tool, have considerable potential for onward use in a variety of clinical and research applications via representation as dimensions of psychopathology.

## Introduction

Advances in personalized psychiatry depend on large-scale biological sampling as well as researchers having ready access to high-quality patient characterization information, including systematic and accurately acquired data on clinical signs and symptoms. The OPCRIT program [1], which in the last 20 years has been used extensively as a patient characterization tool, is suitable for such a role. It contains a checklist constructed from the operational criteria for the major psychiatric classificatory systems, as well as a suite of proprietary algorithms which produce research-quality diagnoses.

Due to the extensive prior use in research and concise structure of OPCRIT, we recently introduced ‘OPCRIT+’ [2] into routine use within a large mental health trust (The South London and Maudsley NHS Foundation Trust – ‘SLaM’). OPCRIT+ is an expansion of the original OPCRIT, incorporating patient history and an increased diagnostic repertoire and sits within SLaM’s electronic health record (ePJS), where all of the trust’s clinical information is stored. OPCRIT+ acts as a data collection and diagnostic device, useable across a broad range of patient settings and from which data suitable for a variety of clinical and research applications are made available.

Although OPCRIT has most commonly been used to produce diagnoses, one potential application of the symptom data systematically acquired on OPCRIT+ will be to generate dimensional representations of psychopathology. In such an approach, a patient’s illness is represented by scores on clusters of symptoms found to occur together in specific patient groups. A number of studies have already used OPCRIT in this manner in psychotic and affective disorders. Using principal components analysis (PCA) or factor analysis, the extracted dimensions have typically been found to represent mania, depression, positive symptoms, disorganization and negative symptoms. Several studies have also compared dimensional against categorical (diagnostic) representations of illness in exploring associations with illness characteristics and clinical outcome measures [3], [4], [5], [6]. All of these reported that a dimensional, or a dimensional and categorical approach combined, was superior to a categorical approach alone. This indicates the considerable research potential offered from the use of the symptom data being recorded with OPCRIT+.

Whilst the introduction of such a tool into routine clinical settings holds considerable promise, there are notable methodological differences between the previous use of OPCRIT and the use of OPCRIT+ in routine clinical care. Typically, OPCRIT has been completed by experienced psychopathology raters reviewing medical notes whereas OPCRIT+ is mainly being completed by junior doctors in busy inpatient units. Therefore, the viability and potential utility of creating dimensional representations of psychopathology from the symptom data being recorded on OPCRIT+ cannot be assumed. In this paper we have set out to examine this. First, we report a PCA which determined the underlying dimensional structure of the symptom data. Next, using component scores, we report on differences between clinical diagnoses in terms of psychopathology represented by these dimensions. Finally, to gain insight into the utility of this approach, we detail the predictive power of component scores, in comparison to clinical diagnosis, for a variety of clinical outcome measures.

## Materials and Methods

### Ethics Statement

All clinical data, stored on the forms used in this analysis, was extracted from ePJS via the ‘Clinical Record Interactive Search’ system (‘CRIS’; [7]) which is a search engine and anonymization portal allowing researchers access to patient data stored on the electronic record. Ethical approval for CRIS as an anonymised data resource for secondary analyses was provided by Oxfordshire REC in 2008 (Reference 08/H0606/71), in accordance with the Declaration of Helsinki, as well as by the Institute of Psychiatry’s Institutional Review Board. Individual patient consent is therefore not necessary for CRIS projects as all data is anonymized at the point of extraction.

### Subjects

Data on 876 patients admitted to SLaM inpatient units between May 2008 and November 2011 were used in this analysis. SLaM operates 68 inpatient units across four main hospital sites. As the introduction of OPCRIT+ within SLaM is an on-going process, we could only use data from units where the form was currently in use; this included: 1 addictions unit, 1 affective disorders unit, 1 eating disorders unit, 1 brain injury unit, 1 psychiatric triage service, 4 forensic units and 8 ‘acute’ wards. For this analysis, ICD-10 diagnosis was assigned by using the closest recorded clinical diagnosis to when the assessment of symptoms with OPCRIT+ was made (mean difference: 82 days, S.D: 322). The distribution of diagnoses and demographic information are detailed in Table 1.

**Table 1:** Distribution of ICD-10 clinical diagnoses and demographic information.

*Diagnosis*	*N (%)*	*Median age*	*Percent male*
**F00–09 Organic, including symptomatic, mental disorders**	**17 (1.9)**	**53**	**70.6**
F06 Other mental disorders due to brain damage and dysfunction and to physical disease	8 (0.9)	49	62.5
**F10–F19 Mental and behavioural disorders due to psychoactive substance use**	**314 (35.8)**	**41**	**65.3**
F10 Mental and behavioural disorders due to use of alcohol	165 (18.8)	45	67.3
**F20–F29 Schizophrenia, schizotypal and delusional disorders**	**292 (33.3)**	**37**	**75.7**
F20 Schizophrenia	200 (22.8)	37.5	78
**F30–F39 Mood (affective) disorders**	**143 (16.3)**	**44**	**60.1**
F31 Bipolar affective disorder	67 (7.6)	47	50.7
**F40–F48 Neurotic, stress-related and somatoform disorders**	**40 (4.6)**	**42**	**80**
F43 Reaction to severe stress, and adjustment disorders	24 (2.7)	40	83.3
**F50–F59 Behavioural syndromes associated with physiological disturbances and physical factors**	**38 (4.3)**	**28**	**0**
F50 Eating disorders	38 (4.3)	28	0
**F60–F69 Disorders of adult personality and behaviour**	**26 (3)**	**39.5**	**65.4**
F60 Specific personality disorders	26 (3)	39.5	65.4
**F70–F79 Mental retardation**	**6 (0.7)**	**35**	**66.7**
F70 Mild mental retardation	5 (0.6)	32	80
Total	876	40	65.9

Rows provide details for all cases within 8 broad ICD ranges (in bold) and underneath each of these the accompanying largest two-digit subgroup within that range.

### Assessments

#### ICD-10 form

Primary (used in this analysis) and secondary ICD-10 clinical diagnoses are recorded on this form. Diagnoses were recorded either at the two e.g. F20 or three-digit level e.g. F20.2. Therefore, for the purposes of this analysis, we compressed all diagnoses into the two digit level.

#### OPCRIT+

Psychopathology present at, or near to, inpatient admission was rated with the ‘Mental State Examination’ section of OPCRIT+ [2]. Only symptom data is detailed in this analysis, as other sections required for OPCRIT+ to produce diagnoses (e.g. ‘History of Presenting Complaint’) were not yet in use. The majority of mental state examinations undertaken within SLaM are done by junior doctors; as such, they were tasked with completing OPCRIT+.

The Mental State Examination section consists of a series of free-text fields corresponding to the standard categories of a mental state examination e.g. ‘Appearance & Behaviour’ under each of which lie the original OPCRIT items e.g. ‘Agitated activity’ and the items unique to OPCRIT+ e.g. ‘Anxiety levels abnormal’. Raters typed their assessments, as a standard part of the clinical documentation process, and then coded observed signs and symptoms as ‘present’. Items not marked as such were considered absent. All doctors received training in the use of the form. OPCRIT has established reliability and validity [8] and OPCRIT+, although only recently developed, has demonstrated substantial inter-rater reliability [2]. OPCRIT+ is available for download via the following link: http://sgdp.iop.kcl.ac.uk/opcritplus/.

#### HoNOS (Health of the nation outcome scales)

The HoNOS instrument [9] contains 12 items measuring behaviour, impairment, symptoms and social functioning, each on a 0–4 scale of severity. A HoNOS ‘total’ score is also produced. The scales form part of the English Minimum Data Set for Mental Health and as such are routinely completed for SLaM patients. Assessments are usually made by nursing staff. HoNOS has demonstrated good reliability [9]. A cut-off point, for HoNOS completion, of 14 days either side of the assessment of symptoms was used (mean difference: 0.46 days, S.D: 5.33), reducing the maximum sample size for analysis using these variables to 452. A further 1.3% of data was missing, which was imputed using the expectation-maximization method.

#### Ward stay form

Duration of inpatient episode was ascertained from the ‘Ward stay’ form. These record admission and discharge dates and are usually completed by administrative staff. For the analysis using this variable, we only used subjects who were admitted to one of seven acute wards, as the duration of stay on many of the other wards e.g. an addictions unit, was likely to be determined primarily by factors other than the presence of symptoms e.g. a predefined period of detoxification. We also only included subjects where the documentation of symptoms with OPCRIT+ was made during the first ward stay of an admission i.e. not if the assessment of symptoms was made on a ward they had been transferred to. However, if a subject was subsequently transferred to another ward, after their initial admission, this subject was included. These factors reduced the maximum number of subjects available for analysis with this variable to 252.

### Statistics

**Figure 1:**
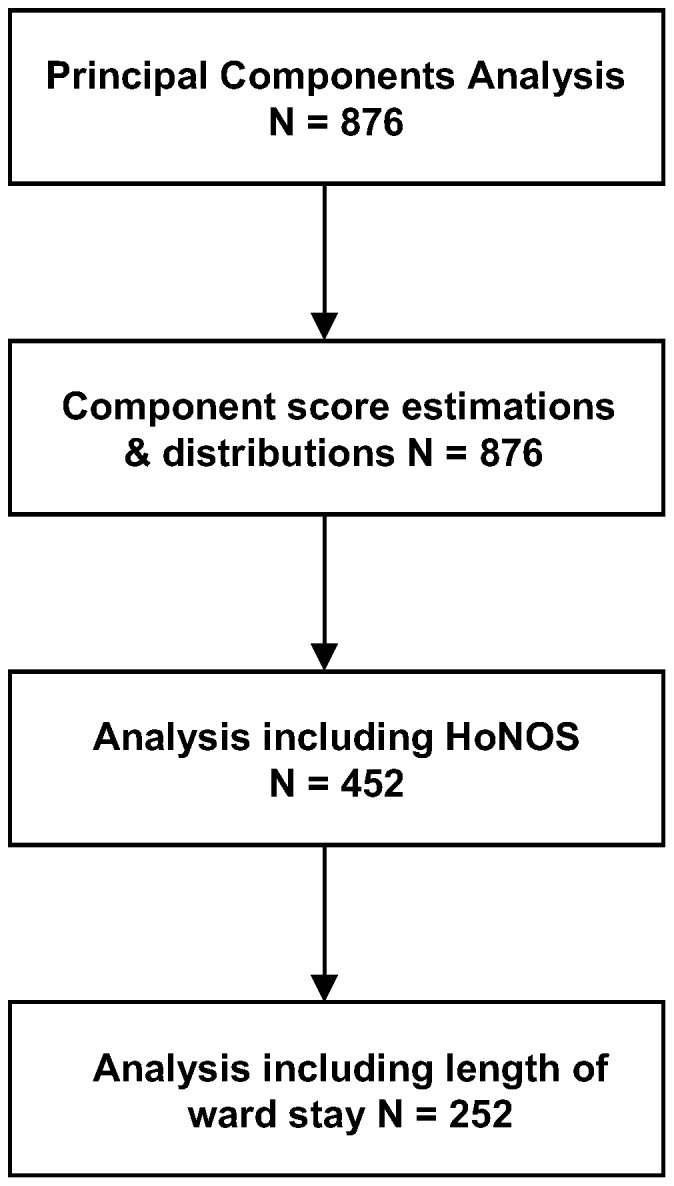
Flow chart detailing the four steps of the analysis and the number of subjects included at each step.

All analyses were undertaken using SPSS version 19. Figure 1 details the various steps in the analysis.

#### Principal Components Analysis (PCA)

Individual OPCRIT+ items were entered into a PCA, a variable reduction technique which maximizes the amount of variance accounted for in the observed variables by a smaller group of variables called components [10]. Items unrelated to phenomenology were excluded e.g. ‘source of rating’, as were items whose variance was near zero i.e. scoring 0 for almost all subjects. In line with previous studies [5], [6], there were several instances where items which had similar meaning were combined to form one variable. These composite items were ‘Restricted or blunted affect’ (combining ‘Restricted affect’ and ‘Blunted affect’), ‘Sleep abnormal’ (combining ‘Initial insomnia’, ‘Middle insomnia’, ‘Early morning waking’ and ‘Excessive sleep’) and ‘Problems with appetite and/or weight’ (combining ‘Poor appetite’, ‘Increased appetite’, ‘Weight loss’ and ‘Weight gain’). A total of 43 items, for each subject, entered the initial analysis as either 0 (symptom not present) or 1 (symptom present). The number of components extracted was based on examination of the scree plot, parallel analysis (a Monte Carlo simulation method) and a requirement that they be interpretable and clinically meaningful. Direct oblimin rotation [11], [12], which allows the extracted components to correlate, was used to aid interpretation.

#### Component score estimation and their distribution within diagnostic classes

Component scores are values indicating a person’s relative standing on a component. These scores can be used to represent severity levels for each subject, on each component, based on a sum of the weighted items which are recorded as being present at the mental state examination e.g. Subject 1 is recorded as having elevated mood+thoughts racing+reduced need for sleep and is therefore more severely manic than subject 2 who is only recorded as having pressured speech. Scores were estimated using the Anderson-Rubin method [13]. Scores are produced based on a group mean centred on 0 with a standard deviation of 1. Scores for components 4–6 were inverted as their initial loadings were negative. Thus, for all components, higher scores represented greater symptom severity. For each component, median scores and the proportion of high scorers (above the upper tertile) were calculated and differences between six of the most frequent diagnoses across the ICD spectrum (F10, F20, F31, F43, F50, and F60) were examined with non-parametric tests of difference (median and chi-squared tests).

#### The relationship of component scores and clinical diagnosis to clinical outcome measures

For each clinical outcome measure (HoNOS ratings and duration of ward stay) we ran three regression models: a full model (Diagnosis+Symptoms; D+S) and two nested models (Diagnosis only; D and Symptoms only; S). Logistic, ordinal or linear regression was used where appropriate. So as to meet cell count assumptions, each model used only a limited number of the more frequent diagnoses (range: 4–9; n: 203–361) with schizophrenia being used as the reference diagnosis in each case. For the same reason, HoNOS items were collapsed into three categories (i.e. 0, 1–2, 3–4) for use in ordinal regression and into a binary rating (i.e. 0, 1–4) for use in logistic regression. Significant models only were compared using the likelihood ratio test. There were four possible conclusions for each clinical outcome measure: 1) D+S>D AND S, meaning a combination of both predictors is best 2) D+S>D but = S, meaning a symptoms only model provides the best fit 3) D+S>S but = D, meaning a diagnosis only model provides the best fit and 4) D+S<D AND S, meaning a combination of the two does not provide a better solution than either alone. In this case, we compared the D and S models separately using Akaike’s Information Criterion [14].

## Results

### Component Structure and Correlations

Inspection of the scree plot and Monte Carlo simulation showed that between 5 and 7 components could be extracted. Examination of the items loading to each component (table 2) suggested that the 6 component solution was superior. These can be considered as representing dimensions of mania, depression, positive symptoms, anxiety, negative symptoms and disorganization. All items had good face validity in relation to their component e.g. ‘Elevated mood’ is a symptom of mania. Primary loadings were all >0.30 with the majority >0.40. Secondary loadings were all <0.25, except in six instances. This solution explained 46% of the overall variance in the data (the sum of the ‘Percent of variance explained’). Four items, ‘agitated activity’, ‘grandiose delusions’, ‘lack of insight’ and ‘inappropriate affect’ were excluded from the final analysis as each one either cross-loaded on more than one component or did not account for a substantial proportion (>0.30) of any components variance.

**Table 2:** Component loadings, after direct oblimin rotation, of the 39 symptoms extracted from the OPCRIT+ checklist.

*Item*	*Component 1* *(Mania)*	*Component 2* *(Depression)*	*Component 3* *(Positive symptoms)*	*Component 4* *(Anxiety)*	*Component 5* *(Negative symptoms)*	*Component 6* *(Disorganization)*	*Communality*
Elevated mood	**.79**	−.03	−.03	.04	−.03	.00	.62
Increased self-esteem	**.77**	−.02	−.07	.03	.01	.07	.57
Thoughts racing	**.74**	.00	−.06	.01	.08	−.06	.57
Excessive activity	**.73**	−.05	−.02	−.05	−.01	−.11	.60
Reckless activity	**.70**	−.06	−.03	−.04	−.16	−.03	.54
Reduced need for sleep	**.68**	.04	.13	.05	.02	.22	.44
Pressured speech	**.65**	−.03	−.07	.05	.13	−.19	.53
Irritable mood	**.38**	.08	.07	−.02	.00	−.11	.19
Loss of energy/tiredness	−.12	**.75**	−.07	−.00	−.11	−.04	.60
Loss of pleasure	−.11	**.74**	−.04	−.06	−.06	−.1	.58
Poor concentration	.13	**.68**	−.08	−.02	−.13	−.12	.54
Dysphoria	.03	**.66**	.13	−.01	.01	−.08	.46
Suicidal ideation	−.04	**.64**	.19	.01	.14	.08	.46
Excessive self-reproach	−.09	**.54**	−.05	−.09	−.12	−.05	.36
Sleep abnormal	.20	**.50**	.06	−.03	.09	.24	.37
Problems with appetite and/or weight	−.05	**.41**	−.03	.03	.03	.14	.20
Altered libido	.26	**.39**	−.09	−.13	−.05	−.02	.28
Abusive/accusatory/persecutory voices	−.02	.03	**.68**	−.01	.00	.06	.46
Third person auditory hallucinations	.04	.02	**.60**	.07	−.00	−.01	.36
Thought insertion	−.04	−.02	**.59**	.06	−.08	−.04	.36
Paranoid/persecutory delusions	.07	−.04	**.58**	.01	.00	−.29	.50
Visual hallucinations	−.01	−.03	**.55**	−.13	.00	.15	.32
Delusions of influence	.02	.05	**.48**	.03	.11	−.26	.33
Hallucination other modality (non-affective)	−.02	−.02	**.47**	−.10	.02	.08	.22
Other (non-affective) auditory hallucinations	−.02	.03	**.44**	.06	−.11	.00	.21
Autonomic arousal symptoms during anxiety	−.04	−.06	−.01	**−.87**	−.00	.01	.74
Recurrent abrupt attacks of severe anxiety	−.04	−.05	−.02	**−.81**	−.00	−.00	.63
Anxiety levels abnormal	−.00	.05	.04	**−.81**	.05	−.01	.67
Prominent, excessive free-floating anxiety	.01	.12	.02	**−.65**	.00	−.02	.48
Negative formal thought disorder	−.04	−.16	.04	.00	**−.83**	.05	.67
Slowed activity	.06	.12	−.04	.03	**−.75**	.17	.58
Restricted or blunted affect	−.08	.22	.06	−.05	**−.57**	−.03	.44
Lack of self-care	.01	.06	.10	.09	**−.37**	−.29	.30
Speech incoherent	.07	.04	−.09	−.04	.01	**−.71**	.52
Positive formal thought disorder	.11	−.04	.04	.00	.13	**−.71**	.56
Speech difficult to understand	.02	.01	−.15	.00	−.11	**−.70**	.53
Bizarre delusions	.00	−.01	.25	.02	.04	**−.44**	.28
Bizarre behaviour	.23	−.17	.12	−.07	−.24	**−.35**	.39
Distractibility	.27	−.09	.16	−.10	−.21	**−.32**	.40
Percent of variance explained	13.5	11	7.5	5.5	5	3.5	

Loadings greater than 0.3 are printed in bold. A six-component solution, with their interpretations, is presented. Item communalities and the percent of variance explained by each component are also presented.

Correlations between component scores, as indicated by Spearman’s rank coefficients, were generally low (table 3). Only a positive correlation between negative and disorganization symptom scores approached a moderate effect size [15].

**Table 3:** Component scores Spearman’s correlations.

	*Mania*	*Depression*	*Positive*	*Anxiety*	*Negative*	*Disorganization*
Mania	1.000					
Depression	.21**	1.000				
Positive	.26**	.08*	1.000			
Anxiety	.19**	.06	.03	1.000		
Negative	−.02	.00	−.01	.00	1.000	
Disorganization	.13**	−.20**	.11**	−.07*	.43**	1.000

* Correlation is significant at the 0.05 level.

** Correlation is significant at the 0.01 level.

### Distribution of Component Scores within ICD-10 Clinical Diagnoses

Median component scores differed significantly between the different diagnoses detailed in table 4 (F06 and F70 were excluded to meet cell count assumptions) for all symptom dimensions except anxiety (Median tests. Mania: X^2^ = 35.263, p<.001, Depression: X^2^ = 48.202, p<.001, Positive: X^2^ = 107.128, p<.001, Negative: X^2^ = 60.261, p<.001, Disorganization: X^2^ = 119.557, p<.001, Anxiety: X^2^ = 5.805, p = 0.326) with the same split occurring in relation to the proportions of individuals scoring about the upper tertile (Chi-squared tests. Mania: X^2^ = 48.614, p<.001, Depression: X^2^ = 27.710, p<.001, Positive: X^2^ = 73.180, p<.001, Negative: X^2^ = 43.465, p<.001, Disorganization: X^2^ = 87.503, p<.001, Anxiety: X^2^ = 10.476, p = 0.063).

**Table 4:** Median and interquartile range Anderson-Rubin component scores and proportion of individuals with high scores (above the upper tertile) as a function of clinical ICD diagnostic category.

*ICD-10 diagnostic category*	*Mania*	*Depressive*	*Positive symptoms*	*Anxiety*	*Negative symptoms*	*Disorganization*
F06 Other mental disorders due to brain damage and dysfunction and to physical disease	−.40/1.44/37	−.47/.99/25	−.48/.59/37	−.39/1.66/37	−.06/.64/50	.13/.50/87
F10 Mental and behavioural disorders due to use of alcohol	−.34/.14/17	−.24/1.22/38	−.49/.10/14	−.37/.15/26	−.43/.18/15	−.48/.28/8
F20 Schizophrenia	−.25/.66/48	−.70/.54/18	.07/1.52/60	−.38/.19/31	−.05/.99/53	.09/1.58/62
F31 Bipolar affective disorder	.07/3.12/67	−.34/1.55/39	−.42/.47/30	−.33/.20/49	−.26/.88/42	−.28/.87/40
F43 Reaction to severe stress, and adjustment disorders	−.32/.24/29	.65/1.62/71	−.33/.99/37	−.37/.55/33	−.38/.84/42	−.43/.50/12
F50 Eating disorders	−.46/.13/13	−.24/1.76/37	−.53/.07/3	−.37/.80/45	−.40/.43/24	−.38/.26/11
F60 Specific personality disorders	−.34/.32/31	.07/1.77/50	.32/.98/69	−.36/.69/46	−.44/.47/19	−.40/.58/23
F70 Mild mental retardation	−.46/.23/20	−.72/.78/0	−.53/1.00/40	−.37/.14/20	−.38/.37/20	−.09/.78/60

Diagnoses listed are the largest two-digit subgroups within each broad ICD range (e.g. F06/F00–09). Figures are in the format of Median/Interquartile range/Proportion of individuals with high scores.

### Association of Component Scores and Clinical Diagnosis to Clinical Outcome Measures

The likelihood ratio test revealed that there were four measures (Overactive, aggressive behaviour; Non-accidental, self-injury; Problems with hallucinations/delusions and Problems with depressed mood) where symptoms alone provided the best fitting model and one measure (Duration of inpatient episode) where diagnosis alone provided the best fit (see table 5). Thus, although the R^2^ was higher in the combined model for all of these measures, removing the diagnoses as a predictor (or symptoms, in the case of ‘Duration of inpatient episode’) did not significantly reduce the fit of the model and thus the smaller model was chosen for reasons of parsimony. ‘Problems with activities of daily living’ was only significantly associated with the symptoms model. R^2^ values in these models was generally low (range: 0.06–0.28). Depression and disorganization were the most frequent significant predictors. Anxiety was not a significant predictor in any of the models. There were a further eight clinical outcome measures which were not significantly associated with any of the three models.

**Table 5:** Diagnosis only (D), symptoms only (S) and models containing both sets of predictors (D+S) and their associations with various clinical outcome measures.

*Clinical outcome measure*	*D*	*S*	*D+S*	*Best model*	*Predictors*
Overactive, aggressive behaviour	.09**	.14***	.17***	S	D, M, Di
Non-accidental, self-injury	.13***	.16***	.19***	S	D, Di
Problem drinking or drug taking	.02	.04	.07	n/a	
Cognitive problems	.02	.04	.06	n/a	
Physical illness or disability problems	.01	.02	.02	n/a	
Problems with hallucinations/delusions	.15***	.28***	.33***	S	P, D, N, Di
Problems with depressed mood	.11***	.16***	.20***	S	M, D
Other mental and behavioural problems	.02	.01	.03	n/a	
Problems with relationships	.01	.01	.03	n/a	
Problems with activities of daily living	.02	.06*	.09	S^b^	N, Di
Problems with living conditions	.01	.02	.03	n/a	
Problems with occupation and activities	.02	.03	.05	n/a	
HoNOS Total	.03	.02	.06	n/a	
Duration of inpatient episode^a^	.25***	.18***	.29***	D	F10, F32, F60, F43, F23

Columns 2–4 report Nagelkerke’s Pseudo R^2^ (^a^adjusted R^2^ where linear regression is used) for each model and overall model significance (*significant at the <0.05 level, **significant at the <0.01 level, ***significant at the <0.001 level). Column 5 details the best fitting model based on the likelihood ratio test (p<0.05) or the non-significance of other models in the comparison^b^. Column 6 details, in descending order of significance, predictors in the best model with a p-value of <0.1. M = Mania, D = Depression, P = Positive symptoms, A = Anxiety, N = Negative symptoms, Di = Disorganization, FXX = ICD10 diagnostic category.

## Discussion

In this analysis, using a newly developed electronic assessment tool (OPCRIT+), we identified a six-component symptom structure underlying the psychopathology recorded in a large, mixed-diagnostic, inpatient cohort. Using component scores to indicate severity, we demonstrated distinct symptom profiles across different clinical diagnoses for five of the six components. Furthermore, these severity scores provided significant predictive value, which was more informative than diagnosis, for a range of clinical outcome measures.

The component structure we extracted is similar to those reported in studies using the original OPCRIT for this purpose [3], [4], [5], [6], [16], [17], [18], [19], [20], [21], [22], [23], [24], [25], [26], [27], [28], [29], [30]. In fact, the five most commonly reported components (or factors) in those studies were also extracted in our PCA: mania, depression, negative symptoms, disorganization and positive symptoms (although the specific OPCRIT items associated with these components varies somewhat across studies). This similarity occurred despite the fact that over half of the patients in our study belonged to diagnostic categories outside the psychotic and affective spectrum, from where cohorts in the other studies were drawn. One notable difference in our component structure however, was the extraction of an ‘anxiety’ component. This occurred due to the additional items in OPCRIT+ allowing the diagnosis of anxiety spectrum disorders.

The extracted components explained 46% of the variance in the symptom data being recorded. This is at the lower end of the range seen in the studies cited above (mean: 52.2% range: 39–71%). There are a number of possible explanations for this. For example, it may be because our PCA contained ratings from a large number of doctors, whereas those in the cited studies typically contained far fewer raters. Alternatively, it could have resulted from the addition of patients whose primary diagnosis was outside the psychotic and affective spectrum and who may have presented with more heterogeneous symptom profiles. Despite this, the successful extraction of an underlying component structure is a vital first step in onward use of the data.

Following the PCA, we created component scores for all subjects to indicate severity levels on each of the six symptom dimensions. We then investigated the distributions of these scores as a function of clinical diagnosis. There were distinct distributions, by diagnosis, for five out of the six components, demonstrated by different median scores and proportions of ‘high-scorers’. Scores on the anxiety dimension did not differ in these respects, indicating that doctors were rating all in-patients as having similar levels of anxiety. Different distributions of symptoms between diagnoses would be expected and support the construct validity of measuring symptom severity in this way. It is notable though, from inspection of the median and inter-quartile range figures, that there was substantial symptom heterogeneity within diagnoses. This variability, in its most extreme form meant that, for example, there were patients with a diagnosis of F10 ‘Mental and behavioural disorders due to use of alcohol’ in the upper and lower 5% of scores on four out of the six dimensions (positive symptoms, mania, depression and anxiety).

We then investigated the predictive power of component scores by following an existing literature whose aim has been to establish the superiority of dimensional, categorical or combinatorial representations of psychopathology. There were five clinical outcome measures where dimensional representations of illness alone provided the best model, whereas there was only one measure where a categorical representation alone was best. There were no measures where a combined approach provided the best solution. The superiority of dimensional over categorical representations of psychopathology, as demonstrated here, is in agreement with other studies which have asked this question using the original OPCRIT [3], [4], [5]; although one study concluded that combinatorial approaches were best [6]. It is important to note however, in relation to the above observations, that we were using ICD diagnoses collapsed to the 2-digit level (due to variation in the way clinical diagnoses were documented). It may be, that at the three digit level or higher (e.g. F10.52), categorical representations of psychopathology would exhibit greater predictive power as well as less symptom heterogeneity.

Despite their overall superiority to diagnosis in this analysis, the predictive value of the component scores, for this set of clinical outcome variables, was only modest (indicated by low R^2^ values and eight measures having no association with the ‘symptoms only’ model). It is therefore important that the utility of this approach in other research realms (e.g. biomarker research) is explored further, particularly as one intended use of the data will be to characterize associated biological and neuroimaging information being gathered in a Bioresource (Biobank) operated by the trust and its partners. It may be that categorical or combinatorial representations of psychopathology are more appropriate for other research areas. Crucially though, via the adoption of OPCRIT+ by SLaM, researchers will now have access to both symptom and diagnosis data recorded in the clinic.

In summary, our analysis has demonstrated that using OPCRIT+, symptom data being routinely recorded across a broad diagnostic spectrum within inpatient settings can be reused to represent severity levels on psychopathological dimensions. This has been achieved despite the very different methodological circumstances between our study and the previous use of OPCRIT for this purpose. Symptom dimensions are applicable across a variety of research and clinical applications and have the potential to add significant explanatory power to many types of analyses.
