# Correction: Harnessing Clinical Psychiatric Data with an Electronic Assessment Tool (OPCRIT+): The Utility of Symptom Dimensions

**DOI:** 10.1371/annotation/d026ac78-2460-40ac-a045-98e95a7b1783

**Published:** 2013-09-10

**Authors:** Philip James Brittain, Sarah Elizabeth Margaret Lobo, James Rucker, Myanthi Amarasinghe, Anantha Padmanabha Pillai Anilkumar, Martin Baggaley, Pallavi Banerjee, Jenny Bearn, Matthew Broadbent, Matthew Butler, Colin Donald Campbell, Anthony James Cleare, Luiz Dratcu, Sophia Frangou, Fiona Gaughran, Matthew Goldin, Annika Henke, Nikola Kern, Abdallah Krayem, Faiza Mufti, Ronan McIvor, Humphrey Needham-Bennett, Stuart Newman, Dele Olajide, David O’Flynn, Ranga Rao, Ijaz Ur Rehman, Gertrude Seneviratne, Daniel Stahl, Sajid Suleman, Janet Treasure, John Tully, David Veale, Robert Stewart, Peter McGuffin, Simon Lovestone, Matthew Hotopf, Gunter Schumann

The last author wishes to add the following to the Funding Statement concerning their funding sources: "Gunter Schuman is also part-funded by the European Union-funded FP6 Integrated Project IMAGEN (Reinforcement-related behaviour in normal brain function and psychopathology) (LSHM-CT- 2007-037286), the FP7 projects ADAMS (Genomic variations underlying common neuropsychiatric diseases and disease related cognitive traits in different human populations) (242257) and the Innovative Medicine Initiative Project EU-AIMS (115300-2), as well as the Medical Research Council Programme Grant "Developmental pathways into adolescent substance abuse" (93558) and the Swedish Research Council (FORMAS)." 

